# Influence of Ocular and Systemic Variables on Intraocular Pressure Change Following Phacoemulsification in Non-Glaucomatous Eyes

**DOI:** 10.18502/jovr.v20.17557

**Published:** 2025-12-08

**Authors:** Ashley H. Yaskanich, Wei Fang, Ibrahim Elwarfalli, Joel R. Palko

**Affiliations:** ^1^West Virginia University School of Medicine, Morgantown, WV, USA; ^2^West Virginia Clinical and Translational Science Institute, Morgantown, WV, USA

**Keywords:** Cataract Extraction, Intraocular Pressure, Phacoemulsification

## Abstract

**Purpose:**

To evaluate the influence of preoperative ocular and systemic variables on intraocular pressure (IOP) change following phacoemulsification cataract extraction with intraocular lens (CEIOL) implantation in eyes without glaucoma.

**Methods:**

This retrospective cohort study included adults who underwent standalone CEIOL at a single academic center between 2017 and 2020. Preoperative demographic, ocular, and systemic health data were extracted from electronic health records using an informatics-based approach and validated by chart review. Eyes with glaucoma or other preoperative ocular conditions known to affect IOP were excluded. Postoperative IOP data were collected for up to 2 years and censored at the time of any subsequent diagnosis or treatment likely to influence IOP. A linear mixed-effects model was used to assess associations between IOP change and preoperative ocular (e.g., IOP, axial length, central corneal thickness) and systemic variables (e.g., diabetes, body mass index [BMI], smoking status), accounting for inter-eye correlation.

**Results:**

A total of 1992 eyes from 1755 patients were included. Higher preoperative IOP ( β = –0.563, *P* < 0.001) and female gender ( β = –0.229, *P *= 0.005) predicted greater postoperative IOP reduction. Longer axial length ( β = 0.201, *P* < 0.001), diabetes mellitus ( β = 0.291, *P *= 0.019), thicker cornea ( β = 0.008, *P* < 0.001), and higher BMI ( β = 0.022, *P *= 0.005) were associated with a relative increase in postoperative IOP.

**Conclusion:**

Both ocular and systemic variables significantly influenced postoperative IOP change following CEIOL in non-glaucomatous eyes. Understanding these associations may improve clinical decision-making and help tailor IOP-related counseling for patients undergoing cataract surgery.

##  INTRODUCTION

Multiple studies have reported a reduction in mean intraocular pressure (IOP) following phacoemulsification cataract extraction with intraocular lens (CEIOL) insertion, both in eyes with and without glaucoma.^[1,2,3,4]^ This IOP-lowering effect has been observed to persist for at least 1 year postoperatively.^[5,6]^ Although the mechanisms by which CEIOL lowers IOP are not definitively established, it is hypothesized that widening of the iridocorneal angle after lens removal and the effects of phacoemulsification energy on trabecular meshwork remodeling may enhance aqueous outflow.^[7,8]^

Several studies have examined ocular variables and IOP change following CEIOL to better understand its IOP-lowering mechanism and to guide clinical prediction of therapeutic IOP reduction.^[9,10]^ A higher preoperative IOP has consistently emerged as the most significant predictor of IOP reduction following CEIOL in glaucomatous and non-glaucomatous eyes.^[5,9,11]^ Preoperative anterior segment metrics such as anterior chamber depth (ACD), lens thickness (LT), combined metrics such as lens position (LP, calculated as ACD + ½ LT), and pressure-to-AC depth ratio (preoperative IOP/preoperative ACD) have been associated with IOP changes after CEIOL.^[9,10][12][13]^ Preoperative axial length (AL), central corneal thickness (CCT), and white-to-white (WTW) distance have shown variable predictive values across studies.^[7,10,12,13,14]^

While numerous studies have examined ocular biometric parameters, the body of research evaluating demographic and systemic health variables in relation to IOP change is less robust. Systemic health variables have been considered primarily in relation to cross-sectional IOP magnitudes. Body mass index (BMI), systemic hypertension, diabetes mellitus (DM), and smoking have been associated with cross-sectional IOP.^[15,16,17,18,19,20]^ The limited number of studies investigating age, DM, and systemic hypertension as predictors of IOP change after CEIOL have reported inconsistent results.^[1,12,21]^

This study used an informatics-based approach to extract data from electronic health records (EHR) for a large cohort without glaucoma. The objective of this retrospective cohort study was to evaluate the influence of demographic and preoperative ocular and systemic health variables on IOP change following CEIOL, with the goal of improving IOP outcome prediction and gaining further insight into mechanisms of underlying post-CEIOL IOP reduction.

##  METHODS

### Data Collection

Ocular and systemic data were automatically extracted from the EHR of consecutive adult patients undergoing phacoemulsification cataract surgery with intracapsular intraocular lens insertion at the West Virginia University Eye Institute from January 2017 to December 2020. The data were collected in accordance with the approved protocol (#2002908592) from the West Virginia University Institutional Review Board, Morgantown, West Virginia, United States of America. Patients with diagnoses of any glaucoma were determined using International Classification of Diseases (ICD)-10 codes, in addition to American Medical Association (AMA) Current Procedural Terminology (CPT) codes related to glaucoma or ocular hypertension treatment before, concurrent with, or up to 2 years after CEIOL. CPT codes associated with glaucoma or ocular hypertension treatment (e.g., filtering surgery, angle surgery, laser trabeculoplasty) were defined as per the AMA CPT Manual from 2017 to 2022 [Supplementary File, Table 1]. Secondary ocular procedures known to influence IOP (e.g., pars plana vitrectomy, retinal detachment repair, keratoplasty, intravitreal steroid injection) were also determined using CPT codes [Supplementary File, Table 2] and chart review when necessary.

Other data collected from the EHR included patient age at the time of surgery, gender, ethnicity, preoperative IOP, IOP measurements at postoperative visits through 2 years, and systemic health variables, including a diagnosis of DM, tobacco smoking status, and BMI. When multiple IOP measurements were recorded during a given appointment, the final applanation tonometry value was used, as this typically reflected the attending physician's measurement. If no applanation value was documented, the first non-applanation measurement was included instead. Intraocular measurements from the Zeiss IOLMaster 700 (ZEISS, Jena, Germany), including AL, CCT, WTW, ACD, and LT, were manually populated for each eye included in the final dataset.

### Manual Validation of Informatics Data

Diagnosis of glaucoma was checked against manual patient chart reviews in 30% of all patients, including 50% of patients labeled with a glaucoma diagnosis. For eyes found to have undergone a procedure other than CEIOL via CPT codes, the procedure type and laterality were verified, and the procedure date was determined through EMR review. Laterality was manually checked in all eyes. Twenty percent of IOP measurements were cross-checked against patient charts.

### Exclusion Criteria

Eyes from patients who had a glaucoma diagnosis or who underwent treatment for glaucoma or ocular hypertension were excluded from the cohort. Eyes with secondary ocular procedures known to influence IOP (e.g., vitrectomy, retinal detachment repair, keratoplasty, intravitreal steroid injection) were excluded if the procedure was performed prior to or concurrent with CEIOL, and postoperative data were censored at the time of the procedure if performed after CEIOL. Eyes undergoing procedures not known to significantly influence IOP in non-glaucomatous eyes (e.g., intravitreal anti-vascular endothelial growth factor [anti-VEGF] injections, YAG capsulotomy, eyelid procedures) were retained.

### Descriptive Data Representation and Statistical Analyses

Descriptive graphs were generated to (1) help specify the model used for the regression analysis and (2) to visualize how IOP changes evolved across the postoperative period according to strata of DM, sex, AL categorized relative to the study median, and ranges of baseline IOP values.

A multivariable linear mixed-effects (LME) model was subsequently used to analyze the association between time (i.e., time elapsed between study entry and each subsequent visit), preoperative variables including baseline IOP, AL, CCT, LP, WTW, DM, gender, age, smoking status, and BMI, and the primary outcome of interest (i.e., postoperative IOP change relative to the baseline IOP values at each visit time). Interaction terms were included to investigate relationships between time and other variables. To account for the correlation between measurements from both eyes of the same patient, laterality was treated as nested within subjects, and a mean value was used for each of the covariates collected from different eyes of the same patient. All statistical analyses were performed using R 4.3.3 (R Core Team, Vienna, Austria), the “lme4” (Version 1.1-35.2), and “rms” (Version 6.8-0) packages.

**Table 1:** Preoperative characteristics of study patients and eyes

**Characteristic**		***n***	**Percentage (%) of total**
Eye laterality			
	OD	1041	52.3 ${}^{*}$
	OS	951	47.7 ${}^{*}$
Sex			
	Male	814	46.40
	Female	941	53.60
Ethnicity			
	White	1604	91.40
	Black	34	1.90
	Hispanic	9	0.50
	Asian	6	0.30
	Hawaiian/Pacific Islander	2	0.10
	Other/unknown	100	5.70
DM			
	Has any DM	832	47.40
	No DM	923	52.60
Smoking status			
	Never	884	50.40
	Former	656	37.40
	Current	215	12.30
		**Mean**	**SD**
Age (years)		67.01	11.02
BMI (kg/m ${}^{2}$ )		31.07	7.07
Baseline IOP (mmHg)		15.56	3.21
AL (mm)		24.11	1.39
CCT (* μ * m)		553.74	41.01
LP (ACD + (0.5 LT))		5.42	0.78
WTW (mm)		12.05	0.42
*Percentage of total eyes; OD, right eye; OS, left eye; DM, diabetes mellitus; BMI, body mass index; IOP, intraocular pressure; AL, axial length; CCT, central corneal thickness; LP, lens position; ACD, anterior chamber depth; LT, lens thickness; WTW, white-to-white.			

**Figure 1:**
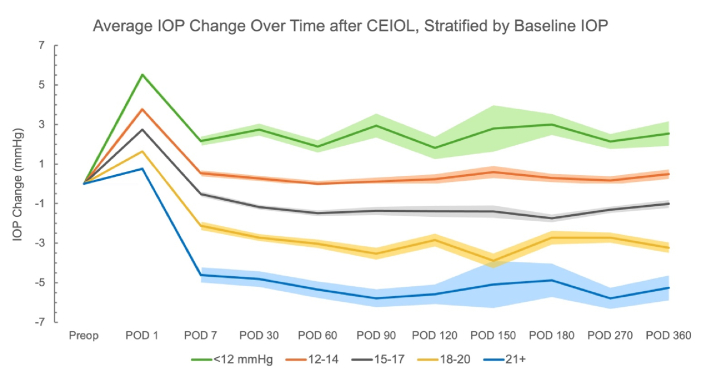
Average change in IOP at postoperative visits, stratified by baseline IOP. Shaded regions represent standard error (SE). SE is not represented until POD 7 for ease of visualization.

**Figure 2:**
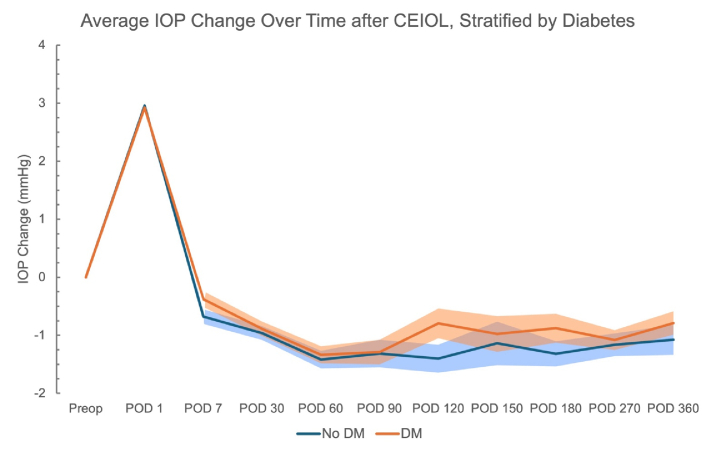
Average change in IOP at postoperative visits, stratified by diabetes diagnosis. Shaded regions represent standard error (SE). SE is not represented until POD 7 for ease of visualization.

**Figure 3:**
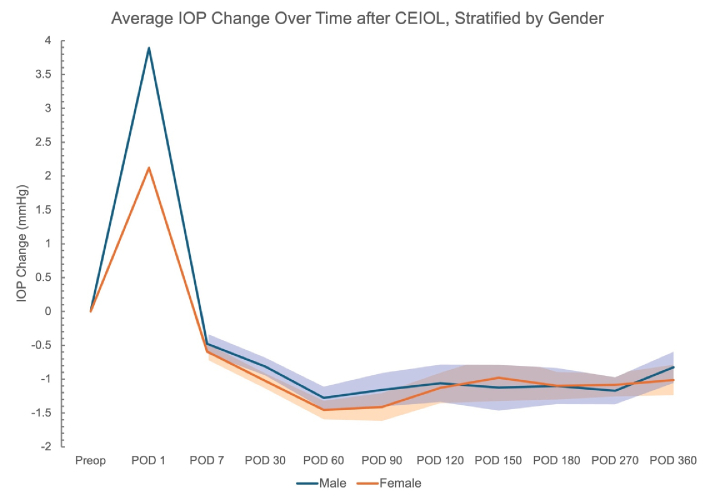
Average change in IOP at postoperative visits, stratified by gender. Shaded regions represent standard error (SE). SE is not represented until POD 7 for ease of visualization.

**Figure 4:**
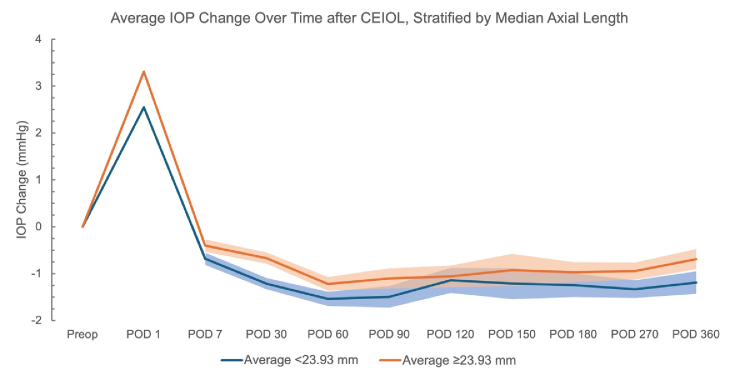
Average change in IOP at postoperative visits, stratified by median axial length. Shaded regions represent standard error (SE). SE is not represented until POD 7 for ease of visualization.

**Table 2:** Linear mixed-effects model analysis of the association between preoperative variables and postoperative IOP change after phacoemulsification cataract surgery

**Parameter**	*** β ***	**± SE**	***P*** **-value**	**95% CI**
Preop IOP	–0.563	± 0.0170	< 0.0001 ${}^{*}$	–0.596 to –0.529
Time	–0.00336	± 0.000465	< 0.0001 ${}^{*}$	–0.00427 to –0.00245
Female sex	–0.229	± 0.111	0.0048 ${}^{*}$	–0.445 to –0.0120
Age	–0.00969	± 0.00506	0.0555	–0.0196 to 0.000227
Former smoker	–0.0308	± 0.184	0.8670	–0.391 to 0.330
Never smoker	0.135	± 0.176	0.4421	–0.210 to 0.481
BMI	0.0223	± 0.00784	0.0045 ${}^{*}$	0.00690 to 0.0376
DM present	0.291	± 0.108	0.0191 ${}^{*}$	0.0784 to 0.504
AL	0.210	± 0.0428	< 0.0001 ${}^{*}$	0.126 to 0.294
CCT	0.00781	± 0.00129	< 0.0001 ${}^{*}$	0.00528 to 0.0103
LP	–0.185	± 0.128	0.1478	–0.436 to 0.0656
WTW	–0.268	± 0.144	0.0627	–0.550 to 0.0142
*P < 0.05; * β *, effect size coefficient; SE, standard error; CI, confidence interval; IOP, intraocular pressure; BMI, body mass index; DM, diabetes mellitus; AL, axial length; CCT, central corneal thickness; LP, lens position; WTW, white-to-white; Adjusted to: Preop IOP = 15.25, Time = 150, Gender = male, Age = 67.17, Smoking = current smoker, DM = present, AL = 23.93, BMI = 30.14, CCT = 552, LP = 5.51, WTW = 12.08.				

**Figure 5:**
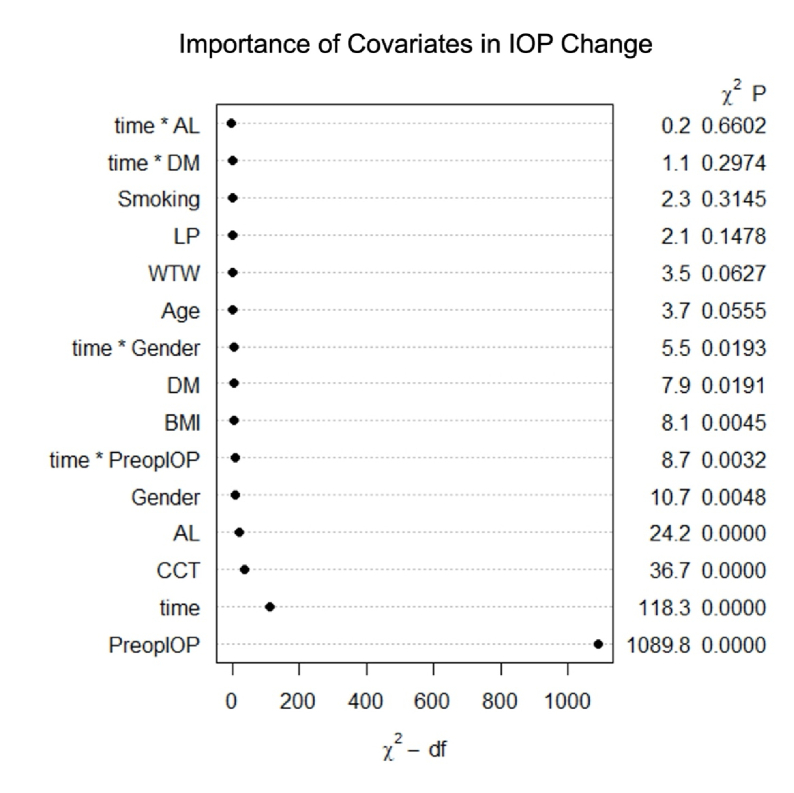
Importance of covariates and covariate-time interactions in postoperative IOP change, ranked by magnitude of likelihood ratio chi-square minus degrees of freedom.

##  RESULTS

Manual validation of the informatics data revealed that of the eyes checked, 91% had a glaucoma status consistent with chart review, and 100% had consistent IOP values. Following application of all exclusion criteria, 1992 eyes from 1755 patients remained for analysis. The number of study eyes retained for follow-up, operationalized as number of eyes seen in clinic in a given postoperative time period, was as follows: 30 days: *n* = 1987 (99.8% of total eyes); 90 days: *n* = 1401 (70.1%); 180 days: *n* = 1061 (53.3%); 360 days: *n* = 488 (24.5%); 450 days: *n* = 541 (27.2%); 630 days: *n* = 449 (22.5%); and 720 days: *n* = 208 (10.4%).

The preoperative characteristics of the 1992 eyes from 1755 patients included in the analysis are shown in Table 1. There was a slight predominance of right eyes and female patients, as well as a high predominance of patients of White ethnicity. The percentage of patients with a diagnosis of DM was 47.3%, and 49.8% of patients were former or current smokers. The mean BMI amongst patients was 31.06 ± 7.09. The mean preoperative IOP was 15.6 ± 3.2 mmHg, and the mean postoperative IOP at 1 year was 14.6 ± 3.1 mmHg.

As expected, mean IOP increased immediately after CEIOL at the postoperative day 1 visit, then decreased over subsequent visit time points.^[22,23]^ On average, eyes within a higher baseline IOP range had a greater absolute IOP reduction at each postoperative visit [Figure 1]. The number of eyes in preoperative IOP strata was as follows: < 12 mmHg: *n* = 162; 12 to 14: *n* = 618; 15 to 17: *n* = 688; 18 to 20: *n* = 392; and 21+: *n* = 126. Data were also stratified by diagnosis of DM and gender [Figures 2 & 3]. On average, eyes from patients without DM had a greater absolute reduction in IOP across the postoperative period [Figure 2]. The average IOP reduction was greater in eyes from female patients across earlier postoperative visit time points [Figure 3]. Eyes with AL below the median value of 23.93 mm had greater IOP reduction on average as compared to eyes with equal to or above that median AL [Figure 4].

The results of the LME model assessing the relationship between preoperative variables and IOP change relative to baseline IOP values following CEIOL are shown in Table 2. Variables used in the model were adjusted to their median values as follows: age (67.17 years), BMI (30.14 kg/m^2^), preoperative IOP (15.25 mmHg), AL (23.93 mm), CCT (552 μ m), LP (5.51), WTW (12.08 mm), time (150 days), gender (male), DM (not present), and smoking status (current smoker). With a 1 mmHg increase in preoperative IOP, the change in IOP decreased by 0.563 (*P* < 0.0001; 95% confidence interval [CI], –0.596 to –0.529). In comparison to male patients, female patients had a postoperative IOP reduction of 0.229 (*P *= 0.0048; 95% CI, –0.445 to –0.0120). The presence of DM was found to increase the change in IOP by 0.291 (*P* = 0.0191; 95% CI: 0.0784 to 0.504). With a one-unit increase in BMI, the postoperative IOP change increased by 0.0223 (*P* = 0.0045; 95% CI, 0.00690 to 0.0376). Age was found to be a marginally significant predictor (*P* = 0.0555), while smoking history was not found to be a significant predictor (*P* = 0.3145). With unit increases in AL and CCT, the change in IOP increased by 0.210 (*P* < 0.0001; 95% CI, 0.126 to 0.294) and 0.00781 (*P* < 0.0001; 95% CI, 0.00528 to 0.0103), respectively. LP (*P* = 0.1478) and WTW (*P = *0.0637) were not found to be significant predictors of postoperative IOP change. With a 1-day increase in time elapsed between study entry and subsequent visit, the change in IOP decreased by 0.00336 (*P* < 0.0001; 95% CI, –0.00427 to –0.00245). Significant interactions with time were found for preoperative IOP (*P *= 0.0032) and gender (*P *= 0.0091), but not for DM (*P *= 0.2974) or AL (*P *= 0.6602). Figure 5 displays variables and interactions in order of importance in postoperative IOP change.

##  DISCUSSION

This retrospective cohort study investigated the relationship between ocular and systemic health variables and IOP reduction following CEIOL in eyes without glaucoma. Consistent with previous studies, higher preoperative IOP was strongly associated with greater postoperative IOP reduction, making it the most significant predictor among the analyzed features [Figure 5].^[1,2,3]^ It is important to acknowledge that this finding may be partially explained by regression to the mean, a phenomenon in which eyes with higher baseline IOP tend to experience a net decrease in IOP over time, while those with lower baseline IOP tend to show a net increase.^[24]^ This study also identified time in the postoperative period as a key predictor of postoperative IOP change, relative to other variables; however, the interaction between preoperative IOP and time played a less prominent role.

Shorter AL was a significant predictor of IOP reduction after CEIOL, as previously reported.^[1,12]^ A leading hypothesis for the mechanism behind the association between AL and post-CEIOL IOP change is the relationship between AL and ACD. Shorter eyes, which often have shallower chambers preoperatively, experience greater anterior chamber deepening after lens removal, potentially improving aqueous outflow and resulting in a larger IOP decrease.^[7,13,25]^ However, LP (ACD + ½ LT), a more direct metric of anterior chamber morphology, was not found to be a significant predictor of IOP change following CEIOL as in prior studies.^[10,13]^ The lack of relationship between LP and IOP change in our study may suggest that the influence of AL on IOP change is less related to anterior chamber morphology. Alternative mechanisms may include differences in the relative effect of phacoemulsification on the trabecular meshwork or in ocular compliance changes following lens removal between eyes of different AL.^[8][10,13][26]^

**This study found that the diagnosis of DM was a significant predictor of increased postoperative IOP. Several cross-sectional studies have associated DM with elevated IOP.^[19,20,21]^ The mechanism behind this association is unknown but may involve increased levels of transforming growth factor β 2 and advanced glycation end-products within the trabecular meshwork of patients with DM, which can impair trabecular meshwork function and aqueous outflow.^[27,28,29]^ These exact mechanisms may contribute to the differences in post-CEIOL IOP change observed in those with and without DM in our study.**

Per unit increase, the effect size ( β in Table 2) of BMI and CCT were both found to be predictive of post-CEIOL IOP, although to a lesser extent than AL and preoperative IOP. It is important to note that, given the larger range of magnitude and variance for BMI and CCT, particularly CCT, their contributions to post-CEIOL IOP change were still relatively high in our LME model [Figure 5]. Corneal thickness is known to influence IOP measurements; however, its role in long-term IOP change after CEIOL remains poorly understood.^[30]^ A higher BMI has been associated with increased IOP in cross-sectional studies, potentially related to orbital fat deposition and elevated episcleral venous pressure.^[31,32]^ CEIOL would not be expected to modulate these distal outflow factors, which may explain why patients with an elevated BMI experience less IOP reduction following CEIOL.

Age was found to be a near-significant predictive variable, while smoking and WTW were not found to be significant predictors of IOP change after CEIOL. The female gender was also associated with a greater IOP reduction following CEIOL. The influence of estrogen on IOP has been previously investigated, but the role of other sex steroids and gender-specific factors in IOP change after CEIOL is unknown.^[33,34]^

This study is limited by the characteristics of the sample eye population and the use of informatics methods. Most patients were of White ethnicity (representative of the regional population, but not the national population), which limited generalizability. Generalizability is also limited by the inclusion of only non-glaucomatous eyes in the analysis. However, the inclusion of only non-glaucomatous eyes allowed for the analysis of predictor variables without the influence of glaucoma-specific variables, including glaucoma subtype, time elapsed since diagnosis, use of glaucoma drops, and glaucoma surgical or laser interventions. Though extensive manual validation of the EHR helped mitigate potential errors in automated data collection, informatics methods may have erroneously categorized or simplified data. For example, IOP measurements used for analysis may have been obtained with varying methods of tonometry. Although infrequent, cases such as sulcus IOL placement could not be reliably identified or excluded unless accompanied by a relevant CPT code (e.g., vitrectomy), limiting the ability to assess their potential influence. Finally, the number of eyes maintained for follow-up at later time points was markedly reduced, although this is not unexpected for the relatively healthy eyes in this cohort.

These findings corroborate prior literature on the influence of preoperative IOP, CCT, and AL on post-CEIOL IOP change, and offer novel insights into less-studied systemic variables, such as DM and BMI. Recognizing predictors of post-CEIOL IOP change may help refine surgical decision-making, especially in cases of glaucoma suspects or ocular hypertension, where adjunctive glaucoma procedures (e.g., angle-based glaucoma surgery) might be considered. Given the potential for sustained IOP reduction, these insights may also contribute to expanding indications for isolated cataract surgery in this subset of patients. Future research directions to improve generalizability include performing similar analyses in eyes with glaucoma. Additionally, the use of machine learning models may allow an individualized prediction of the magnitude of IOP change following CEIOL.^[35]^

In summary, we found that both ocular and systemic variables influenced postoperative IOP changes after CEIOL in non-glaucomatous eyes.

##  Financial Support and Sponsorship

This project was supported in part by the West Virginia Clinical and Translational Science Institute.

##  Conflicts of Interest

None.
